# The relationship between psychosocial risk factors, burnout and quality of life among primary healthcare workers in rural Guangdong province: a cross-sectional study

**DOI:** 10.1186/s12913-019-4278-8

**Published:** 2019-07-03

**Authors:** Joseph Obiri Asante, Meng Jie Li, Jing Liao, Yi Xiang Huang, Yuan Tao Hao

**Affiliations:** 1 Department of Biostatistics and Epidemiology, School of Public Health, Sun Yat-sen University, 74# Zhongshan 2nd Road Building 20, Guangzhou, Guangdong People’s Republic of China; 2 Department of Health Policy and Management, School of Public Health, Sun Yat-sen University, 74# Zhongshan 2nd Road Building 20, Guangzhou, Guangdong People’s Republic of China

**Keywords:** Quality of life, Psychosocial risk factors, Medical practice, Primary healthcare, Community healthcare, Burnout

## Abstract

**Background:**

Healthcare workers are often exposed to stressful working conditions at work which affect their quality of life. The study investigated the relationship between psychosocial risk factors, stress, burnout, and quality of life among primary healthcare workers in general medical practice in Qingyuan and Chaozhou cities in Guangdong province.

**Method:**

The cross-sectional study was conducted in 108 primary health facilities including 36 community health centers (CHCs) across two developing cities in Guangdong province. A total of 873 healthcare workers completed the questionnaires. Quality of life was evaluated using The World Health Organization Quality of Life Questionnaire (WHOQOL-BREF) and psychological risk factors were evaluated by the Copenhagen Psychosocial Questionnaire (COPSOQ). General quality of life and the quality of life domains were transformed into a score range from minimum 0 to 100 maximum. Higher scores indicated better quality of life and vice versa. Significant associations were verified using multiple regression analysis.

**Results:**

Poor quality of life was observed in 74.6% of healthcare workers surveyed. General poor quality of life was significantly higher among workers who reported higher burnout (Beta = − 0.331, *p* < 0.001). In addition, workers with high levels of burnout, unmarried workers and female workers had a higher possibility of physical health. A greater risk of poor psychological health was observed among workers with high burnout, poor sense of community and those with lower educational levels. Workers who lacked social support, those with fewer possibilities for development had increased probability of poor quality of life in the social domain. Poor quality of life in the environmental domain was observed among workers who were dissatisfied with their jobs and workers with low salaries.

**Conclusions:**

Primary healthcare workers in developing cities in China have a highly demanding and strained working environment and poor quality of life. Reducing job stress and improving work conditions may ultimately improve the well-being of primary healthcare workers.

## Background

Quality of life (QoL) is a very complex concept that influences the level of commitment and productivity at work regardless of profession. In primary healthcare delivery, QoL can contribute to positive outcomes for healthcare providers and patients [1]. Healthcare workers’ state of happiness and well-being affect their attitude toward patients, ability to interact with patients, ability to disseminate information to patients, quick service delivery, and therefore the provision of quality medical care [2]. On the other hand, poor well-being may result in poor performance, absenteeism, low commitment, and inappropriate patient care.

In China, challenges that confront the health sector like inadequate resources and the shortage of healthcare workers especially in the rural areas if not handled properly might hinder the achievement of the sustainable development goals (SDGs) [3]. The United Nations General Assembly in 2015 adopted the SDGs with the 2030 Agenda for sustainable development i.e. eradicating poverty and ensuring sustainability of the planet. The SDG goal number 3 focuses on the promotion of well-being and healthy living for all people of all ages. The new healthcare reform plan which came into effect in 2009, places a premium on the pivotal role of primary healthcare workers including healthcare professionals at the community healthcare centers (CHCs). The plan emphasizes the need for equitable, affordable and accessible health care to the public by strengthening its primary health delivery system [4–6]. The government recognizes primary healthcare workers as major stakeholders and considers them crucial to the success of the new health reforms. Nevertheless, many medical professionals prefer to work in developed cities with better working conditions, adequate resources, and opportunities for career development. On the other hand, there are other health personnel working in poor conditions in rural China. In addition to low salaries and restricted opportunities for promotions in rural China, limited resources and shortage of skilled professionals creates challenges in the provision of quality healthcare services due to job load and overtime work, which ultimately lead to lower efficiency, job stress and negative health effects among healthcare workers [7–9]. Negative outcomes of job stress are associated with illness, decreased capacity to perform, reduced initiative drive, reduced efficiency, reduced work interest, job dissatisfaction, absenteeism, lack of concern for the organization, increased staff turnover, poor well-being, and the decline in overall quality of care [10]. These factors have been recognized as financially costly to any healthcare organization.

One of the most important work environment issues in China is psychosocial risk factors which pose a great challenge to health and quality of life. A previous study indicates that poor working environment can adversely affect productivity, health, and workers’ well-being [11]. In addition, psychosocial risk factors may result in work-related burnout, cognitive stress symptoms and job dissatisfaction [12]. In order to improve productivity and increase efficiency in the primary healthcare delivery, it is imperative to explore the psychosocial needs of health providers. However, few studies have investigated adverse psychosocial factors at work and the impact of these factors on quality of life of primary healthcare workers in rural China. The purpose of this study has been to investigate the relationships between psychosocial risks factors arising from work, job stress, burnout and impact on perceived quality of life among primary health workers in rural China.

## Methods

### Design and sampling

The study was a cross-sectional study conducted between July and October 2017 in public hospitals of relatively underdeveloped cities in Southern Guangdong province. A purposive sampling method was used to select two developing cities in Guangdong province namely Qingyuan and Chaozhou. These two cities are classified as rural or developing cities based on their level of economic development (i.e. per capita income) and other social criteria such as life expectancy and literacy rates. In this study, the term rural that applies to the geographic context was defined as communities with most residents being farmers, often referred to as ‘county’. Considering the fact that hospitals are divided into three different levels in these cities i.e. provincial, municipal and county, multi-stage random sampling was employed in selecting hospitals in each city. In all, 18 hospitals were selected at the different levels in each city, eventually, 108 hospitals including 36 CHCs were included in the study. We used simple random sampling to determine the study sample of healthcare workers from each hospital. Ten healthcare workers from each hospital were randomly selected from the hospitals based on the willingness of respondents to participate in the study. This was made possible after a list of all the potential respondents was prepared initially and each potential respondent accorded specific number. From this population, random samples were chosen using the random number generator software. Data were collected through self-administered questionnaires. Respondents were asked to fill and submit anonymous questionnaires. The inclusion criteria were as follows: healthcare workers aged 18–60 years, working in public hospitals. We focused on the age range 18–60 years because this is the typical age range of the workforce in the population studied*.* Respondents with major health conditions and illness were excluded from the study.

### Respondents

The respondents were divided into three categories based on their professions. The categories were physicians, nurses/midwives and other healthcare workers (HCWs) (e.g. pharmacists, biomedical scientists, physiotherapists, health assistants, and community health workers). A total of 1000 questionnaires were administered, 500 questionnaires for each city. The response rate was 87.3%, accurate and completely filled questionnaires were considered valid. Incomplete questionnaires were not included in our study. Due to the anonymous nature of the survey, written informed consent was not obtained nonetheless, verbal consent was obtained from each participant and this was approved by the Ethics Committee of Sun Yat-sen University.

### Instrument

A structured questionnaire was used and it came in three parts. The first part of the questionnaire measured respondents’ demographics such as age, gender, marital status, educational background etc. This was followed by a second part which was the Chinese translation of the validated Copenhagen Psychosocial Questionnaire (COPSOQ). COPSOQ is a robust, broad and all-encompassing instrument used to measure psychosocial risk factors at work in its entirety [13, 14]. The Chinese version had been tested in various sectors and different professions in China and demonstrates good validity and reliability (i.e. Cronbach’s alpha > 0.7 for most scales) [15, 16]. The COPSOQ has 25 scales with five distinctive domains namely; demands, influence and development, interpersonal relations and leadership, further parameters and outcome scales [14, 17]. All the questionnaire questions had corresponding responses measured on a 1~5 Likert scale. The scores were transformed into minimum 0, 25, 50, 75 and maximum 100. The domain values were calculated by taking the average of the number of scale items in a domain.

The final section of the questionnaire was the Chinese version of the WHO quality of life scale (WHOQOL-BREF) which consisted of 26 items [18]. The WHOQOL-BREF has four domains: physical, psychological, social and environmental domains [19]. The first two questionnaire items assessed the overall quality of life and general health. The first question on the WHOQOL-BREF, “how would you rate your quality of life?” measures the general quality of life and the second question, “how are you satisfied with your health?” measures general health. Good quality of life was categorized by responses; “very good” and “good” while poor quality of life was categorized by responses; “neither poor nor good”, “poor” and “very poor”. With regards to the four domains, the mean score of the individual items within each domain was used to calculate the domain score. Each domain scores were transformed into a score range from lowest 0 to the highest 100. The higher the scores the better the quality of life in that domain and vice versa. The final scores for each domain were classified into three quartiles. Workers who had higher scores (i.e. upper quartile) had a better quality of life while lower scores (i.e. lower quartile) were considered to have a poor quality of life.

### Variables

#### Independent variables

In this study, we considered socio-demographics as independent variables. Socio-demographics considered were age, gender, marital status, salary, educational level, profession, department, work experience, seniority (i.e. senior[> 10 yrs], middle [6-10 yrs] or junior staff [< 5 yrs]), type of contract (fixed-term contract or no fixed contract), employment status (i.e. full-time [6-8 h/d] or part-time[less than 6 h, normally 2-3 h/d]) and overtime (*i.e* working at least 1 time per month on weekends or holidays, or at least 1 time per week evenings [after 18:30] or nights [before 5:00] or working at least 1 time per week from home/outside of the office /at customers). In addition, the following psychosocial risk factors were also considered as independent variables; variables measuring demands at work, influence and development at work, interpersonal relations and leadership, further parameter and job strain.

#### Dependent variables

General poor quality of life, poor QoL in the physical, psychological, social and environmental domains were considered as dependent variables. There were 26 individual items on the WHOQOL-BREF, the first two items examine the overall quality of life and general health, and the other 24 items examine well-being in four key domains i.e. physical health-seven items, psychological health-six items, social domain-three items, and environmental domain-eight items. The physical health domain include items on daily activities, functional capacity, mobility, pain and sleep. Psychological health domain measures self-esteem, self-image, mentality, memory and concentration, negative thoughts and positive attitudes. The social relationship domain measures social support, personal relationship, and sex life. The environmental health domain include safety, living physical environment, general environment (air pollution and noise etc), financial resources, health and social services and transport.

### Statistical analysis

Data were analyzed descriptively using means (M) and standard deviations (SD) then with independent sample t-test. The independent sample t-test was used to examine the differences in COPSOQ scales and QoL domains among male and female groups. Forward stepwise multiple regression analysis was performed to test the association between the independent variables and the generally poor quality of life and each of its domains. General poor quality of life and poor QoL domains were treated as dependent variables whiles psychosocial risk factors were treated as independent variables. Statistical significance was set at *p*-value < 0.05. All Data were analyzed using SPSS software (version 20).

## Results

### Socio-demographic characteristics of respondents

A total of 873 respondents correctly filled and submitted the questionnaires out of a total of 1000 questionnaires circulated, representing 87.3% response rate. Table 1 shows the socio-demographic characteristics of healthcare workers. There was just about the same representation of men and women in the study. Majority of the healthcare workers were married, and had obtained college diplomas or above with average monthly salary of 501.39 ± 320.18 US dollars. Approximately 72.9% of health workers were dissatisfied with their salaries. 35.4% of respondents indicated that money is what comes to mind when we mention when quality of life, again 58.7% of the respondents indicated that money encourages them to perform well however, 49.83% indicated that they are not motivated by their facilities. When asked to rate their state of health between 0 and 10, where 0 indicates worse health and 10 indicates best state of health, 70.79% of respondents rated their state of health above 5.

**Table 1:** Summary of socio-demographics of the study population

Variable Total	Overall (*N* = 873)	Percentage (%)	SD
Age (years)			
18~25	132	15.1	
26~30	237	27.2	
31~40	414	47.4	
41~50	84	9.6	
51~60	6	0.7	
Gender			
Male	444	50.9	
Female	429	49.1	
Marital status			
Single	168	19.2	
Married	678	77.7	
Divorced & Others	27	3.1	
Education			
Elementary school	6	0.7	
High school	12	1.4	
College Diploma	459	52.6	
Degree	345	39.5	
Others	51	5.8	
Profession			
Physicians	591	67.7	
Nurses/midwives	216	24.7	
Other HCWs	66	7.6	
Salary (USD)			
< $200	39	4.5	
$200–399	306	35.1	
$400–599	276	31.6	
$600–799	192	22.0	
$800–999	41	4.8	
> $1000	18	2.0	
Management Categories (Levels)			
Senior Staff	141	16.2	
Middle level staff	303	34.7	
Junior staff	429	49.1	
Employment status			
Full-time	777	89.0	
Part-time	96	11.0	
Supervisor for other employees			
Yes	192	22.0	
No	681	78.0	
Fixed employment contract			
Yes	750	85.9	
No	123	14.1	
Quality of life			
Poor	651	74.6	
Good	222	25.4	
Physical Domain			
Poor	609	69.8	
Good	264	30.2	
Psychological domain			
Poor	489	56.0	
Good	384	44.0	
Social domain			
Poor	160	18.3	
Good	713	81.7	
Environmental domain			
Poor	456	52.2	
Good	417	47.8	
Salary USD (M ± SD)	501.39		319.82
Total work experience [years](M ± SD)	10.70		6.48
Work experience on current job[years] (M ± SD)	8.07		6.35

*M* Mean, *SD* standard deviation N- total number

### Healthcare workers’ psychosocial risk factors and quality of life

The Cronbach’s alpha was used to assess the reliability of the WHOQOL-BREF and the COPSOQ. The overall Cronbach’s alpha values for the WHOQOL-BREF and COPSOQ were 0.929 and 0.788 respectively. The results show good reliability of the two instruments used (Table 2). Table 3 shows the score values and standard deviations for the COPSOQ scales and the QoL domains.

**Table 2:** Cronbach’s alpha for COPSOQ and WHOQOL-BREF

Domain	Number of items	Cronbach’s alpha
COPSOQ scales	22	0.788
Demands	4	0.896
Influence and development	4	0.834
Interpersonal relationship and leadership	8	0.888
Job strain	5	0.679
QoL Domains	24	0.929
Physical Domain	7	0.785
Psychological Domain	6	0.799
Social Domain	3	0.706
Environmental Domain	8	0.862

**Table 3:** Psychosocial risk factors and quality of life domains scores

COPSOQ scales & QoL Domains^a^	Number of items	Mean ± SD
COPSOQ Scales		
Demands		
Quantitative demands	4	59.79 ± 16.49
Emotional demands	3	56.50 ± 19.80
Demands for hiding emotions	2	73.93 ± 21.69
Work conflicts and privacy	5	54.04 ± 24.18
Influence and development		
Influence at work	4	35.48 ± 20.80
Degree of freedom at work	1	32.82 ± 31.09
Possibilities for development	4	52.17 ± 18.57
Meaning of work	3	53.29 ± 24.59
Interpersonal relations and leadership		
Predictability	2	35.56 ± 26.91
Role-clarity	4	61.96 ± 17.15
Role-conflict	2	51.85 ± 23.29
Quality of leadership	4	41.60 ± 27.90
Social support	4	60.77 ± 18.15
Feedback	2	54.39 ± 18.99
Social Relations	2	63.54 ± 15.56
Sense of community	3	55.29 ± 13.50
Further parameters		
Job insecurity	4	39.07 ± 24.87
Job Strain (Outcome scales)		
Intention to leave	2	22.34 ± 21.16
Job satisfaction	7	49.61 ± 16.98
Burnout	6	58.47 ± 17.18
Cognitive stress	3	66.69 ± 20.31
General health	1	67.83 ± 20.95
QoL Domains		
Physical domain	7	43.06 ± 10.57
Psychological domain	6	47.13 ± 14.46
Social domain	3	58.51 ± 15.75
Environmental domain	8	46.53 ± 17.02

*QoL* Quality of life, *SD* standard deviation

^a^ Possible transformed score for each scale between 0(minimum) and 100(maximum)

**Fig. 1:**
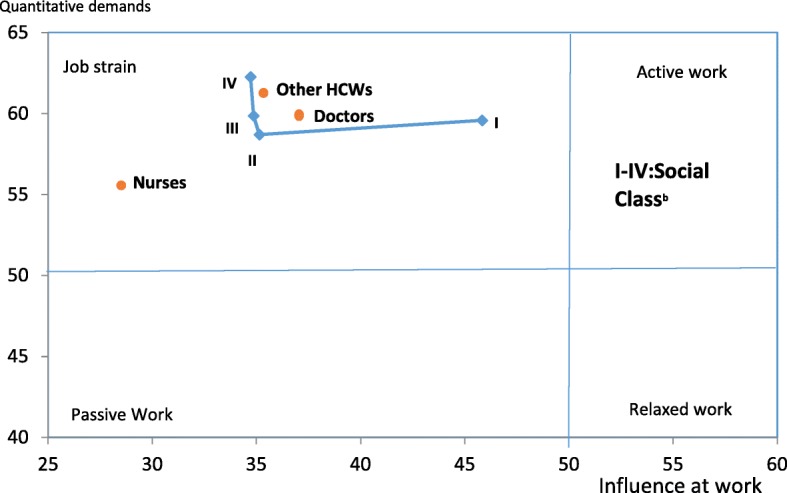
Job strain as measured by the COPSOQ questionnaire. I-IV: social class. ^b^ Social classes were categorized using job grades and salary levels of health workers. Other HCWs: Other healthcare workers

Respondents reported high scores for scales; the demands for hiding emotions (73.93), cognitive stress symptoms (66.69), quantitative demands (59.79) and burnout (58.47) weighed on a scale of 100. The higher scores for these scales indicate a challenging and highly strained work environment. Moreover, the following scales recorded lower scores; intention to leave (22.34), the degree of freedom at work (32.82) and job satisfaction (49.61). This suggests that workers were not satisfied with their jobs, they had less freedom at work however they had no intention of early retirement or quitting. In addition, the four QoL domains recorded low scores ranging from 58.51 for the social domain to 43.06 for the physical domain. Male workers reported significantly higher scores for the scales; quantitative demand, emotional demands, work conflicts, and burnout. Higher scores for demands and burnout are considered detrimental to health and well-being. On the other hand, female workers reported better quality of life, higher job satisfaction, and better social support compared to male workers (Table 4). However, female respondents had higher cognitive stress symptoms. Figure 1 gives a picture of the work environment among health care workers. The findings show that the work environment is very demanding and highly strained for all health professionals included in the study.

**Table 4:** Psychosocial risk factors and Quality of life comparison among male and female healthcare workers

Scales	Male	Female	*P* value^a^
	M ± SD (*n* = 444)	M ± SD (*n* = 429)	
COPSOQ Scales			
Demands			
Quantitative demands	61.82 ± 16.86	57.69 ± 15.88	< 0.001
Emotional demands	60.08 ± 19.95	52.79 ± 19.02	< 0.001
Demands for hiding emotions	75.84 ± 21.01	71.94 ± 22.26	0.008
Work conflicts and privacy	58.41 ± 23.11	49.51 ± 24.50	< 0.001
Influence and development			
Influence at work	39.78 ± 20.76	31.03 ± 19.95	< 0.001
Degree of freedom at work	34.63 ± 31.34	30.94 ± 30.83	0.080
Possibilities for development	54.64 ± 18.62	49.61 ± 18.23	< 0.001
Meaning of work	55.69 ± 25.42	50.82 ± 23.53	0.003
Interpersonal relations and leadership			
Predictability	39.44 ± 26.43	31.56 ± 26.90	< 0.001
Role-clarity	63.22 ± 17.47	60.66 ± 16.76	0.028
Role-conflict	54.73 ± 23.34	48.86 ± 22.93	< 0.001
Quality of leadership	42.78 ± 28.81	40.38 ± 26.98	0.205
Social support	59.42 ± 18.87	62.17 ± 17.34	0.025
Feedback	55.20 ± 19.53	53.57 ± 18.44	0.203
Social Relations	63.24 ± 1.54	63.85 ± 14.53	0.567
Sense of community	55.47 ± 14.21	55.10 ± 12.77	0.687
Further parameters			
Job insecurity	39.95 ± 24.24	38.15 ± 25.56	0.286
Strain (effect Outcome scales)			
Intention to leave	23.48 ± 21.17	21.15 ± 21.15	0.104
Job satisfaction	47.71 ± 17.99	51.57 ± 15.68	0.001
Burnout	59.86 ± 18.04	57.03 ± 16.18	0.015
Cognitive stress	65.20 ± 18.66	68.24 ± 21.85	0.027
General health	68.04 ± 22.36	67.62 ± 19.46	0.768
QoL Domains			
Physical domain	43.72 ± 11.72	42.38 ± 9.22	0.060
Psychosocial domain	46.39 ± 15.14	47.89 ± 13.72	0.124
Social domain	58.03 ± 16.51	59.01 ± 14.97	0.358
Environmental domain	46.52 ± 18.27	46.53 ± 15.69	0.997
Self - evaluation of Qol	50.00 ± 21.43	54.20 ± 15.97	0.001

^a^ Independent sample t-test; *SD* standard deviation, *n* Number of respondents, *M* Mean

### Impact of psychosocial risk factors on quality of life and QoL domains

Table 5 shows the associations between psychosocial risk factors and poor quality of life. The risk of a respondent recording generally poor quality of life was significantly higher for workers who reported higher levels of burnout (Beta = − 0.331, 95% CI [− 0.310, − 0.205], *p* < 0.001), those dissatisfied with their jobs (Beta = 0.230, 95% CI [0.121, 0.259], *p* = 0.001) and those with job insecurities (Beta = − 0.085, 95% CI [− 0.087, − 0.009], *p* = 0.015) after controlling for sex and age. There is also a high prevalence of generally poor quality of life among workers who described their work as less meaningful (Beta = − 0.272, 95% CI [− 0.198, − 0.109], *p* = 0.08), poor social relations with management or colleagues (Beta = 0.191, 95% CI [0.100, 0.241] p < 0.001) and poor sense of community (Beta = − 0.175, 95% CI [− 0.244, − 0.093], *p* < 0.001).

**Table 5:** Forward stepwise regression analysis of predictors of general poor quality of life^a^

Dependent variable	Scales	Beta	95% CI for *B*		R^2^	*P* value
			Lower bound	Upper bound		
Quality of life	Burnout	−0.331	−0.310	− 0.205	0.309	< 0.001
	Job satisfaction	0.230	0.121	0.259		0.001
	Meaning of work	−0.272	− 0.198	− 0.109		0.008
	Intention to leave	− 0.150	− 0.137	− 0.046		< 0.001
	Job insecurity	− 0.085	− 0.087	− 0.009		0.015
	Social relations	0.191	0.100	0.241		< 0.001
	Sense of community	−0.175	−0.244	− 0.093		< 0.001

Statistical significance *p* < 0.05; R^2^, Proportion of the variance explained by the model. ^a^General poor quality of life was defined by the general question “How do you classify your quality of life” (good = 0, poor = 1)

In the quality of life domains, respondents who recorded high levels of burnout were at risk of having poor physical and psychological health. Also associated with an increased chance of poor physical health were single workers, workers with less degree of freedom at work and among workers with poor role clarity. In addition, workers who reported a non-existent sense of community, poor social relations, workers who had high emotional demands and those with lower educational level were more likely to experience poor psychological health. Respondents found to demonstrate poor quality of life in the social domain were workers with a lack of social support, workers who showed fewer possibilities for career development and those with a poor quality of leadership. Moreover, workers who were dissatisfied with their jobs, salaries, had no opportunities for career development and those with work conflicts were more likely to have a poor quality of life in the environmental domain (Table 6). The recorded variances for the regression models were; physical domain (R2 = 0.67), psychological domain (R2 = 0.72), social domain (R2 = 0.62), and environmental domain (R2 = 0.61).

**Table 6:** Forward stepwise regression analysis of risk factors impacting on poor quality of life in the physical, psychological, social and environmental domains

Dependent variables	Scales	Beta	95% CI for *B*		R^2^	*P* value
			Lower bound	Upper bound		
Physical Health	Burnout	−0.570	−0.228	− 0.117	0.673	< 0.001
	Degree of freedom	−0.521	−0.136	− 0.062		< 0.001
	Marital status	−0.469	−9.670	−4.031		< 0.001
	Role clarity	0.455	0.082	0.203		< 0.001
Psychological health	Sense of community	−0.698	−0.345	−0.185	0.716	< 0.001
	Education	0.237	0.664	4.125		0.008
	Social relations	0.401	0.089	0.223		< 0.001
	Emotional demands	0.272	0.035	0.154		0.003
	Burnout	−0.258	−0.148	−0.019		0.012
Social Domain	Possibility for development	−2.849	−0.921	−0.498	0.617	< 0.001
	Quality of leadership	1.454	0.188	0.409		< 0.001
	Social support	1.512	0.342	0.734		< 0.001
Environment Domain	Role conflicts	−0.798	−0.366	−0.233	0.607	< 0.001
	Emotional demands	0.445	0.082	0.192		< 0.001
	Salary	−0.450	−0.026	−0.013		< 0.001
	Work conflict	−0.326	−0.155	− 0.044		0.001
	Predictability	0.594	0.091	0.177		< 0.001
	Job satisfaction	−0.353	− 0.225	− 0.051		0.002
	Intention to leave	−0.302	− 0.118	− 0.039		< 0.001
	Possibilities for development	−0.460	−0.196	− 0.090		< 0.001
	Degree of freedom	0.344	0.032	0.115		0.001
	Quality of leadership	−0.336	−0.120	−0.028		0.002

*: Statistical significance *p* < 0.05; R^2^, Proportion of the variance explained by the model. Lower scores (i.e. lower quartile) for each of the four domains were considered as poor quality of life

## Discussion

The well-being of every healthcare worker is crucial for quality health care provision, for that reason, this study investigated the association of risk factors and outcomes on quality of life. High score values for quantitative demands at work and low scores for influence at work suggest a demanding and highly strained work environment. This situation may be as a result of increasing aging population, pressure on limited resources and the shortage of health professionals at the rural areas resulting in an increased workload, job stress, depression, and reduced productivity. It is not surprising that the majority of the workers were dissatisfied with their jobs looking at the work conditions they have to deal with daily. Moreover, it has been reported that Chinese physicians are dissatisfied with their jobs and work conditions [20]. Job dissatisfaction is a major predictor of intention to stay on the job and therefore has an influence on job turnover [21]. In addition, Male workers reported higher demands at work, burnout and poor quality of life than women. These findings are consistent with the previous study that suggest that men take on more workload negatively affecting their health and well-being [22]. Furthermore, workers reported high scores for quantitative demands and cognitive stress symptoms but lower degree of freedom at work, consistent with Karasek stress model.

There was a significant association between burnout and poor quality of life, as well as physical and psychological health after controlling for age and gender. This suggests that workers with high burnout are at risk of experiencing poor quality of life. This shows clearly that psychosocial job-related outcomes can adversely affect the quality of life [23]. In the US, the number of physician with burnout symptoms has increased over the years especially among specialties in emergency care compared to other workers in the US [24]. Currently, the cumbersome bureaucracy healthcare workers have to deal with in order to perform their daily task in addition to work pressures, insecurity at work, increased number of hospital visits by an aging population and modern lifestyle changes can lead to exhaustion in healthcare workers which can be detrimental to physical and psychological well-being. The proper task distribution and work organization can positively influence the well-being of healthcare workers. In present-day China, recurrent violence at work, the strained doctor-patient relationship, poor sleeping habits may have deleterious effects on the health and psychological well-being of healthcare workers [20]. This study compares favorably with a study that revealed that medical staff across different countries were stressed and strained [25]. Again, a number of studies have established that physical and mental disorders impact psychological well-being of healthcare workers, placing emphasis on stress, burnout, depression and physical exhaustion [26–30]. In addition, previous studies have shown an association between psychosocial risk factors and quality of life among healthcare workers [23, 31].

Furthermore, 74.6% of healthcare workers reported poor quality of life. Again, quality of life was positively associated with job satisfaction. This result suggests a highly strained and demanding work environment that undermine the well-being of healthcare workers. Therefore, improving job satisfaction may translate into a better quality of life for workers and improve staff retention.

According to our study, single workers and those with less degree of freedom at work tend to have poor physical health. Poor physical health is characterized by frequent bodily pains, lack of energy, burnout, low concentration, and sleeping problems etc. These symptoms can lead to absenteeism, sick leaves and intention to quit [32, 33], leading to high job turnover that may affect the organization financially. There are various studies that argue that work organization, task allocation and pressures at work all affect physical health [31, 34, 35]. Moreover, workers with emotional challenges, poor sense of community, poor social relations and those with less education tend to exhibit poor psychological health. Psychological and mental disorders among healthcare workers may lead to absenteeism and low productivity [36]. Furthermore, workers with less possibility for development, poor quality of leadership and lack of social support exhibited greater probability of poor quality of life in the social domain. Individuals’ personal problems at home may interfere with work life and likewise, individuals’ work challenges such as lack of support from colleagues and superiors, lack of promotional opportunities, weak leadership may spill over into other areas of personal life. The study demonstrates that disgruntled workers, low salaried workers, those with a lack of opportunities for development and those with intentions to quit are likely to exhibit a poor quality of life in the environmental domain. This may reflect job insecurity, inadequate working conditions including remuneration and motivation. A great deal of literature has established that satisfaction with remuneration has a negative association with job turnover [37–40]. Likewise, a report from a previous study reveals a significant association between better working conditions and an improvement in the quality of care [41]. Accordingly, by improving the well-being of healthcare workers may offer significant benefits to health institutions looking for improvement in employee retention, increased productivity, self-confidence and commitment. Identifying risk factors and initiating solution strategies taking into consideration the well-being of healthcare workers may reduce inefficiencies, cost, and frequently experienced errors. Health facilities and stakeholders should implement interventions that may benefit the well-being of healthcare workers in order to improve productivity and quality of care.

The strengths and limitations of this study cannot be overlooked. One major strength of the study is the inclusion of a reasonably large number of different healthcare professionals and the inclusion of many different health facilities. In addition, the study had a high response rate. Additionally, the study was conducted in developing areas in China, adding evidence to literature in this field of research. There were limitations to this study. First, the study used a cross-sectional design, which is not appropriate to assess the direction of causation, therefore an in-depth study should be conducted in the future to explore other factors that could influence the QoL of healthcare workers. Secondly, although the sample was representative enough, the data are not nationally representative and therefore, results cannot be overgeneralized to all healthcare workers in China. Further longitudinal studies in several cities in China would provide a deeper understanding of psychosocial factors impacting on QoL in China.

## Conclusion

The work environment was demanding and highly strained as a result, healthcare workers exhibited poor quality of life. There was an association between psychosocial risk factors and poor quality of life. The prevalence of burnout is highly associated with poor quality of life, as well as its physical and psychological health. Job dissatisfaction was associated with poor well-being of healthcare workers. This study also demonstrated a greater risk of psychological health among workers with a poor sense of community and those with low educational level. Finally, this study revealed that workers with lower salaries were associated with poor quality of life in the environmental domain. It is important for managers of health facilities to initiate strategies to improve working conditions and reduce burnout at work, ultimately improving the well-being of primary healthcare workers.

## Data Availability

Data supporting the findings of the study will be made available upon request.

## Abbreviations

95% CI: 95% Confidence interval
CHCs: community health centers
COPSOQ: Copenhagen psychosocial questionnaire
d: day
GDP: Gross domestic product
HCWs: Healthcare workers
M: Mean
NBSC: National Bureau of Statistics of China
QoL: Quality of life
SD: Standard deviation
SDGs: Sustainable development goals
SPSS: Statistical Package for the Social Sciences
US: United States
USD: United States Dollar
WHO: World Health Organisation
WHOQOL: WHO Quality of Life
WHOQOL-BREF: Abridged WHO Quality of Life
yrs: Years
